# Editorial: Second or foreign language learning and cognitive development

**DOI:** 10.3389/fpsyg.2023.1354329

**Published:** 2024-01-04

**Authors:** Dingfang Shu, Jinfen Xu, Hui Zhang, Zhen Tian

**Affiliations:** ^1^Shanghai Center for Research in English Language Education, Shanghai International Studies University, Shanghai, China; ^2^School of Foreign Languages, Huazhong University of Science and Technology, Wuhan, Hubei, China; ^3^School of Foreign Languages, Nanjing Normal University, Nanjing, Jiangsu, China

**Keywords:** second language (L2) acquisition, foreign language (English) learning and teaching, cognitive development, language teaching, language learning

Research on bilingualism has shown that acquiring a second language enhances a learner's executive function and metalinguistic awareness within the cognitive development domain (Bialystok, 2001; Bialystok and Luk, 2012; Kroll and Bialystok, 2013). Further investigation is necessary to understand the impact of individual differences on the learning process and its result, including the influencing factors like age, gender, gender, first language, learning style, input and feedback types, and teaching methods. Perhaps more crucially, however, is the potential for the learning process to interact with learners' metalinguistic, affective, cognitive, and metacognitive abilities (Dörnyei, 2009; Bylund and Jarvis, 2011; Gass and Mackey, 2015; VanPatten and Williams, 2015).

A total of 29 manuscripts were submitted on the Research Topic. A total of 15 have been accepted, which fall into five broad categories: (1) the interaction between L1 and L2; (2) second or foreign language learning and cognitive controls; (3) second or foreign language learning and social skills and empathy; (4) second or foreign language learning and metacognitive skills; and (5) teaching methodology and L2 and foreign language learning.

One paper in particular, “*Australian English listeners' perception of Japanese vowel length reveals underlying phonological knowledge*,” by Yazawa et al., examines how native speakers of Australian English, who typically emphasize vowel length compared with most other English varieties, perceive Japanese vowel length contrasts. In a forced-choice study, twenty monolingual Australian English speakers were asked to rank the Japanese long and short vowels based on their resemblance to their native vowel categories. The findings indicated a general tendency for Australian English long and short vowels (such as/i:, I/as in “heed,” “hid”) to be classified as Japanese long and short vowels (e.g.,/ii, i/). This contrasts with the literature-reported categorization of all Japanese vowels as tense by American English listeners, regardless of length (e.g.,/ii, i/as both “heed”). The result is consistent with a feature-based speech perception approach.

Research on the shared-dialect effect, which suggests that raters who share a candidate's dialect may provide higher scores on English speaking examinations, was conducted by Xu et al.. Oral performance in the recounting task of the computer-based English Listening and Speaking Test was evaluated by raters proficient in Cantonese and Mandarin. No statistically significant interaction was found between the raters' and candidates' dialects, nor were there any significant variations in the ratings given by either group in the quantitative data. The understanding and scoring process of raters were influenced by their awareness and familiarity with accents, according to the qualitative data.

Wang D. et al. investigate the efficacy and diversity of translation strategies employed by Chinese English as a foreign language (EFL) learners when addressing light verb constructions (LVCs), a significant distinction between Chinese and English, in a different study titled “Walking out of the light verb jungle: Exploring the translation strategies of light verb constructions in Chinese–English consecutive interpreting”. The study examines the methods used by 66 Chinese EFL learners to interpret 12 target LVCs using a theory-driven, context-based interpreting problem. The outcomes demonstrate the typical structural trends in LVC translation as well as the overall preferences for strategy selection among Chinese EFL learners. Additionally, the study reveals a positive relationship between vocabulary knowledge and the acceptability rates of LVCs, indicating the necessity of integrating constructional teaching into EFL instruction.

The research paper titled “*Non-adjacent dependency learning from variable input: investigating the effects of bilingualism, phonological memory, and cognitive control*” by Verhagen and de Bree delves into L2 learning and cognitive control. It sheds new light on the correlation between bilingualism and statistical learning, and compares the effects of consistent and variable input on statistical learning in both monolingual and bilingual children and adults. The study also investigates whether phonological memory and cognitive control play a role in potential group differences. The results indicate that bilinguals have a limited advantage in statistical learning, which is not consistently linked to enhanced cognitive abilities associated with bilingualism.

In recent years, there has been a surge in research on the relationship between emotion and L2 learning, with a particular focus on social skills and empathy. One such study, “*Understanding foreign language writing anxiety and its correlates*” by Li, conducted a quantitative meta-analysis of 84 effect sizes from 22 primary studies to investigate the connections between foreign language writing anxiety and its high and low-evidence correlates. The study revealed moderate correlations between foreign language writing anxiety and writing self-efficacy and performance, as well as moderately positive effects with listening, speaking, and reading anxiety. Additionally, the study found that age and language proficiency have significant moderating effects. The findings have important pedagogical implications, which were discussed based on the results.

Wang H. et al.'s article titled “*Unpacking the relationships between emotions and achievement of EFL learners in China: Engagement as a mediator*” explores the connections between learners' emotions, such as foreign language enjoyment (FLE), foreign language classroom anxiety (FLCA), and foreign language learning boredom (FLLB), and engagement, as well as their English achievement. The study involved 907 English as a foreign language (EFL) learners from a university in China who completed an online questionnaire, and structural equation modeling was used to test the hypothesized relations among the variables. The results showed correlations between learners' FLE, FLCA, and FLLB, and that learners' engagement mediated the relationships between their emotions and English achievement. The study provides evidence for the mechanism underlying the relationships between emotions, engagement, and achievement, and sheds light on EFL teaching and learning at the tertiary level in China.

A third article that falls into this category, *Measuring Chinese English-as-a-foreign-language learners' resilience: development and validation of the foreign language learning resilience scale* by Guo and Li, aimed to develop the Foreign Language Learning Resilience Scale (FLLRS) to measure the psychometric scale reliability and validity of foreign language learning resilience in Chinese English-as-a-foreign-language contexts. Data was collected from 313 Chinese college students, and the FLLRS was validated based on reliability and validity tests. The FLLRS consisted of three factors: ego resilience, metacognitive resilience, and social resilience, all contributing to foreign language learning resilience. Metacognitive resilience had the highest path coefficient, followed by social resilience and ego resilience. The validated scale could advance knowledge in second language acquisition regarding the factors that affect foreign language learning resilience.

Second or foreign language learning and metacognitive skills have been of great interest among researchers. In a study by Qin et al., entitled “*Validation of metacognitive strategies in writing and their predictive effects on the writing performance of English as foreign language student writers*,” the metacognitive writing strategies of EFL college students in China were examined through a survey and a writing test. The study utilized exploratory factor analysis and confirmatory factor analysis to analyze the data, and multiple regression analysis was employed to understand the predictive effects of metacognitive strategies on writing performance. The findings suggest that writing instruction can enhance students' awareness and ability to acquire metacognitive writing strategies, particularly those related to planning, monitoring, and evaluating.

Wang's study, “*Text memorization: an effective strategy to improve Chinese EFL learners' argumentative writing proficiency*,” explored the impact of text memorization strategies on the argumentative writing proficiency of EFL learners in China. The study focused on the text memorization process and the strategies used by learners to enhance memorization. Thirty-three Chinese English majors participated in seven text memorization tests, a pre-test, and a post-test to evaluate their memorization outcomes and writing proficiency before and after memorizing seven model English writings. Additionally, twelve top scorers in the memorization tests were interviewed. The results indicated that text memorization significantly improved learners' writing proficiency. Moreover, a new system of text memorization strategies was developed to assist scholars and teachers in enhancing the writing skills of EFL learners.

Peng and Bao's article, “*Effects of reasoning demands triggered by genre on Chinese EFL learners' writing performance*,” examined the impact of cognitive complexity on the writing performance of advanced Chinese EFL learners in two different genres: expository writing and argumentative writing. The study involved 76 EFL learners who completed two writing tasks with varying levels of reasoning demands. Multiple measure indices, including lexical complexity, syntactic complexity, accuracy, fluency, and cohesion, were used to assess the differences in production dimensions between the two tasks. The results indicated that cognitive complexity significantly enhanced lexical complexity, clausal complexity, and cohesion, but there was a trade-off effect for phrasal and clausal structures within syntactic complexity. The findings of this study have important implications for the sequencing and design of L2 writing tasks.

The success of foreign students' academic and life skills in the Northern Cyprus region is heavily reliant on the importance given to Turkish language teaching. “*Teaching the Turkish language to foreigners at higher education level in northern Cyprus: an evaluation based on self-perceived dominant intelligence types, twenty-first century skills and learning technologies*” by Kurt and Güneyli aimed to investigate how college students use learning technology, 21st-century skills, and perceive intelligence categories in learning a foreign language. The study utilized purposeful and convenience sampling, selecting the institution with the largest number of international students in Northern Cyprus. The results indicated a statistically significant correlation between 21st-century skills and foreign language-learning technology usage, highlighting the importance of modern methodologies and social learning in foreign language education.

Zhao and Huang's article, “*A comparative study of frequency effect on acquisition of grammar and meaning of words between Chinese and foreign learners of English language*,” investigated the impact of frequency on L2 vocabulary acquisition. The study explored the frequency effect on the acquisition of grammar and meaning of alphabetic words between Chinese learners of hieroglyphic language and foreign learners of alphabetic language. The results indicated that mother tongue type may not be the factor causing differences in grammar and meaning acquisition of vocabulary, while exposure frequency of vocabulary plays a determining role. Furthermore, learner types, language types, frequency, and part of speech of a word have an interaction effect on word acquisition. The study sheds light on the importance of frequency in L2 vocabulary acquisition and highlights the need for tailored teaching methods to facilitate this process.

Meng et al.'s article, “*Cognitive diagnostic assessment of EFL learners' listening barriers through incorrect responses*,” utilized Cognitive Diagnostic Models (CDMs) for bugs, or Bug-CDMs, to diagnose EFL learners' listening barriers through incorrect responses. The study found that Bug-GDINA was the optimal model, and semantic understanding and vocabulary recognition were the most prevalent barriers. The findings demonstrate the feasibility of using Bug-GDINA to diagnose listening barriers from incorrect responses.

The majority of research on collocations in L2 acquisition and cognitive psychology has focused on phonographic languages, giving scant attention to ideographic languages such as Chinese and Japanese. In “*The lexical processing of Japanese collocations by Chinese Japanese-as-a-Foreign-Language learners: an experimental study by manipulating the presentation modality, semantic transparency, and translational congruency*,” Song et al. investigated the processing of Japanese collocations by 36 Chinese Japanese-as-a-Foreign-Language learners. The study manipulated presentation modality, semantic transparency, and translational congruency in a lexical judgment task. The results indicated longer reaction times for auditory presentation than visual presentation, and longer reaction times for high semantic transparency and congruent translation in auditory presentation. These findings support the dual-route model of Japanese collocational processing and demonstrate that presentation modality, semantic transparency, and translational congruency have an impact on processing.

He and Gao's study, “*Explicating peer feedback quality and its impact on feedback implementation in EFL writing*,” investigated the impact of peer feedback quality on EFL students' feedback implementation in argumentative writing tasks. The researchers developed a measuring scale with two dimensions to assess feedback quality, including accuracy and revision potential. The results indicated that feedback accuracy was at a medium level, while revision potential was at a low level, with accuracy having a stronger predictive power on implementation. Furthermore, feedback quality had the strongest predictive power when feedback features and focus were considered. The study highlights the importance of training students to provide and implement high-quality feedback marked by good accuracy and high revision potential in future instructions.

For future studies on second or foreign language learning and learners' cognitive development, it would be valuable to explore how the learning process, and learning multiple foreign languages, either simultaneously or consecutively, can impact learners' emotional and cognitive development, and how the new development, in turn, affects the learning process and outcome. This research can provide insights into the relationship between language learning and cognitive development, as well as the potential benefits of multilingualism. Additionally, it may be interesting to investigate how individual differences, such as age, gender, and learning styles, affect this relationship. Understanding the impact of language learning on cognitive development can inform language education and help educators design more effective language learning programs.

## Author contributions

DS: Writing—review & editing. JX: Writing—review & editing. HZ: Writing—review & editing. ZT: Writing—review & editing.
